# The importance of enworlded selfhood for understanding chronic pain-related suffering

**DOI:** 10.1007/s11017-026-09747-4

**Published:** 2026-03-03

**Authors:** Fredrik Svenaeus

**Affiliations:** Centre for Studies in Practical Knowledge, Södertörn University, 141 89, Huddinge, Sweden

**Keywords:** Phenomenology, Chronic pain, Suffering, Mood, Selfhood, Lifeworld

## Abstract

In the research and literature on pain-related suffering, a difference has recently been made between suffering processes of the minimal self in contrast to the hardships of the narrative self. This article proposes that an in-between layer of selfhood needs to be distinguished and studied to understand pain-related suffering in a more thorough manner: the enworlded self. Pain-related suffering is a complex phenomenon involving mood-related processes and everyday challenges on at least three different levels: lived bodily experiences, lifeworld matters, and questions about personal identity. These three different levels correspond to three connected aspects of layered selfhood: the minimal self, the enworlded self, and the narrative self. By using a published memoire focusing on chronic pain experiences, this article attempts to enlighten the middle ground of enworlded selfhood and also identifies two different regions of the lifeworld in which the damaging and alienating experiences take their toll: the work-world and the family-world. In addition, a third region of the lifeworld is identified: the patient-world, a region which is formed and expands as the chronic-pain sufferer searches for medical-professional advice and assistance. By addressing the three different layers of selfhood and how they interact in the everyday life of the patient, chronic-pain suffering can be better understood and, also, possibly alleviated by taking appropriate measures that fit the attuned pattern of the pain experiences of each individual.

## Introduction

Since at least the 1980s there has emerged a growing consensus in health care and medical research that chronic pain gives rise to types of suffering which cannot be explored by ways of biomedicine only but need to be addressed by considering the lived experience of the suffering person (also referred to as ‘self’). Selfhood questions can only be explored by studying the first-person aspect of what it means, in this case, to suffer from chronic pain. Health-care professionals and researchers adopt not only a biomedical third-person perspective, but also a second-person perspective, in attempting to understand the suffering experienced by patients. That is: they do not only study the brains and bodies of chronic-pain patients, but ask the patients about their experiences, do interviews with them, and use pain-scales and questionnaires to better understand their life and how to be able to help them. Notwithstanding the significant breakthroughs in neurophysiological understanding of the pathways and working mechanism of pain made recently [1], this focus on the lived experience of pain-related suffering is perhaps the most important development of pain research since the turn of the century [2].

Such a development is also in line with the definition made by The International Association for the Study of Pain as early as 1979, that pain is to be understood as “an unpleasant sensory and emotional experience associated with, or resembling that associated with, actual or potential tissue damage” [3]. However, although IASP has continued to update the exact phrasing of their pain definition over the years, they have not yet developed any definition of *pain-related suffering* to address the questions of how suffering may relate to the experience of (chronic) pain but still connote a thicker and different concept in this context. Since chronic pain, according to medical and statistical investigations, afflicts as much as a fifth of the world population, this is an urgent task [1].

Attempting to investigate these issues, a systematic review and meta-analysis of scientific articles proposing a definition/conceptualization of pain-related suffering has recently been published in the journal *Pain* by Noe-Steinmüller and colleagues [4]. The sixty articles referred to in the review—which explicitly defined pain-related suffering—were found in journals as diverse as psychology, palliative medicine, medical ethics, nursing, philosophy, sociology, psychiatry and gerontology, revealing how urgent and widespread the need is for a deeper understanding of the phenomenon. Noe-Steinmüller and colleagues aimed to systematize their review by not only identifying eight (obviously overlapping) *dimensions* of pain-related suffering—social, physical, personal, spiritual, existential, cultural, cognitive and affective—but also by suggesting a definition of the concept itself. Using qualitative methods and machine-learning tools the authors put forward the following definition of pain-related suffering: “a severely negative, complex, and dynamic experience in response to a perceived threat to an individual’s integrity as a self and identity as a person” [4, p. 1444].

## The influence of Cassell

The suggested definition reflects Eric J. Cassell’s early idea about characterizing the nature of the suffering experienced by ill persons in addition to pain and bodily symptoms only:

Suffering occurs when an impending destruction of the person is perceived; it continues until the threat of disintegration has passed or until the integrity of the person can be restored in some manner. It follows, then, that although it often occurs in the presence of acute pain, shortness of breath, or other bodily symptoms, suffering extends beyond the physical. Most generally, suffering can be defined as the state of severe distress associated with events that threaten the intactness of the person [5, p. 32].

While this quote is taken from the second edition of Cassell’s book *The Nature of Suffering and the Goals of Medicine*, the author expressed the core of this definition as early as 1982 in a journal article with the same title as the book [6]. Over the years, his idea that suffering consists not only in pain and other bodily symptoms, but also in a perceived threat to the integrity/intactness of the person has become very influential, and, to my mind, rightly so.

Notice that according to Cassell’s definition, pain as such does not qualify as suffering until it has reached a level of intensity and endurance that breaks apart the lives of patients by threatening their “integrity” or “intactness”. The key concept here is “person”, which for Cassell involves many different issues that he does not tie together neatly by means of any philosophical theory of personhood, but nevertheless gives many examples of and excellent discussions about in his book. Cassell chooses the concept of “person” instead of “self”, because he thinks issues of selfhood are somehow “self-concerned” and exclude social and cultural issues [5, p. 33]. However, Cassell is careful to avoid any charges of dualism; instead, he puts matters like this:

Persons have personality and a character, a lived past, a family, a family’s lived past, culture and society, roles, associations with others, a political dimension, activities, day-to-day behaviors, an existence below awareness, a body, a secret life, a believed-in future, and a transcendent spiritual dimension. The importance of these features for understanding suffering is that each can be affected by illness and become a source of suffering if the integrity of the person is thereby disrupted [5, p. 150].

As we have seen, Noe-Steinmüller and colleagues try to get hold of the bodily-felt dimensions of pain-related suffering by stressing that it is “a severely negative, complex, and dynamic experience” [4, p. 1444]. They then add: “in response to a perceived threat to an individual’s integrity as a self and identity as a person”, bringing in a distinction between selfhood and personhood not found in Cassell’s definition [4, p. 1444]. Noe-Steinmüller and colleagues then proceed to address not only personhood but also selfhood in their definition, a move possibly carried out to stress the physical and affective dimensions of suffering in addition to the six other much more person-centred ones—social, personal, spiritual, existential, cultural and cognitive—that they have identified in the literature about pain-related suffering [4]. I will return to this possible distinction between different layers of self/personhood below.

## The minimal self in contrast to the narrative self

Despite these efforts to slightly modify Cassell’s characterization, the definition of chronic-pain related suffering made by Noe-Steinmüller and colleagues has already been charged with a tendency to over-personalize the concept. Timothy H. Wideman and Peter Stilwell with colleagues criticize Noe-Steinmüller and colleagues for neglecting a firm connection between suffering and the pain experiences as such, and, also, for not including non-human animals, preverbal children, and individuals with diseases or disabilities who lack higher cognitive capabilities [7, 8]. It is debatable whether the “integrity as a self and identity as a person” formulation found in Noe-Steinmüller and colleagues [4] solves these problems. However, it should be noted that the dissatisfaction with Cassell’s model can be found already in a focus article written and published in *The Journal of Pain* by Stilwell and colleagues a couple of years before the review article of Noe-Steinmüller with colleagues in *Pain* was published [2].

In this article, Stilwell with colleagues notice the influence of Cassell’s proposal in the literature on pain-related suffering and welcome its focus upon life narratives of suffering persons. What they find lacking is a firm grounding of the concept in the experiences of pain itself, which could lead to suffering in cases of individuals who do not have the capacity to think and/or imagine what the pain in question will mean for their personal identity and future prospects in life. Surveying the literature, they notice four key attributes (central truths) which they think must be considered in developing a definition of pain-related suffering: 1) pain and suffering are inter-related, but distinct experiences; 2) suffering is a subjective experience; 3) the experience of suffering is characterized by a negative affective valence; and 4) disruption of one’s sense of self is an integral part of suffering [2].

To support the intuition that not only persons who have developed language and other advanced cognitive skills—such as time consciousness and narrative comprehension of their own standing in relation to others—may suffer, the authors claim that the disruption of selfhood addressed by Cassell could not only be suffered by what is most often referred to as the “narrative self” [9, 10], but also by what is known in the philosophy of mind as the “minimal self” [11–13]. Stilwell and colleagues put it like this:

Pre-reflective self-experience involves a tacit level of selfhood or “mineness” of experience, often referred to as the “minimal self”. The minimal self is the foundation for the narrative (reflective) self that develops over time [2, p 734].

Every creature that is conscious in the sense of being emotionally aware of its existence has a minimal self. This does not imply self-consciousness in the sense of understanding that one exists as a person in relationship to other persons, it only implies undergoing *experiences*, including feelings of being in pain which *hurt* one’s being [14]. Stilwell and colleagues want to expand the disruptive element of pain-related suffering to include not only the narrative-self/person but also the minimal self which can *feel* this threat to its existence even in cases when the creature/individual in question is lacking narrative personhood. They consider this claim to be validated by descriptions given by persons who have found themselves in situations of *overwhelming* pain and later returned to and reflected upon what it was like (when asked by pain-researchers) [2, 8].

This distinction between the minimal self and the narrative self in experiences of pain-related suffering is likely what Noe-Steinmüller and colleagues are trying to get at by the phrase: “an individual’s integrity as a self and identity as a person” in the previously mentioned review article and definition proposal [4]. Stilwell with colleagues do not propose an exact definition of (chronic) pain-related suffering—instead they state four key-attributes—but the idea of making an explicit difference between the minimal and the narrative self clearly represents a step forward in comparison with Cassell’s person-centred (narrative-self dependent) categorization. They also give many different examples of what the perceived threat to the intactness/integrity of the minimal self in pain-related suffering looks like by quoting and systematizing interviews made with 12 chronic-pain patients [8]. Here are some examples:

*All I was, was like this giant existence of pain … I couldn’t move … it felt like I couldn’t breathe … I couldn’t even access like any part of my brain that was thinking based. All I could focus on was pain … I just couldn’t even get to that point, like where I could cope with what was happening and like use my internal self-talk to be on my side. It’s like that voice was completely missing and gone. And it was only the emotions … I just went backwards, like I went back into the embryo.* (Participant 1)

*You feel like you’ve died as a person … Because pain has just overwhelmed everything … it’s like the pain overrides common sense … analytical abilities were completely gone … It’s almost like your brain shuts down and you’re just in the pain, and the pain is consuming you … an overwhelming sense of loss of self … I’m just this ball of pain. I’m not even a person.* (Participant 5)

*It’s all-consuming … the hardest one* (pain experience) *took my mind … I wasn’t there … I couldn’t advocate for myself* (in the emergency department) *… the pain owned me … I found that it’s the feeling of powerlessness ultimately … and I find it’s terrifying … I wasn’t in control of my body. My body was in control of me … It* (pain) *owns me … An articulate person becomes something different … I wasn’t in control in any way, shape or form.* (Participant 10)

*There was no sense of time … I couldn’t have told you what day of the week it was. I couldn’t tell you what time of day it was. I couldn’t tell you whether it was night or day …* (I felt) *trapped and not moving forward.* (Participant 6)

In contrast to the damage done to the integrity and wholeness of the narrative self (person) in chronic pain-related suffering (focused upon by Cassell), here is witnessed the terrible incapacitating nature of physical pain in and by itself. It would be strange and perhaps even unethical to deny that what these persons are trying to describe is suffering, a kind of suffering which can also be felt by individuals lacking the capacity to comprehend and express this suffering in the form of language.

Stilwell with colleagues attempt to comprehend the suffering done to and felt by the minimal self by stressing three related things: a disrupted sense of agency, a disrupted sense of bodily ownership and awareness, and a disrupted experience of time [8]. I think this analysis is to the point and comes very close to what I have myself in earlier studies characterized as illness suffering in the form of bodily *alienation* with pain as a major example [15, 16]. However, as the authors of the article note themselves, there is a lot of contextual space between the minimal and the narrative self which needs to be covered if one is to reach a full understanding of chronic-pain suffering [8].

In order to accomplish this, I will now introduce a third middle-ground version of the layered self, connecting the minimal to the narrative: *the enworlded self*, making explicit connections to the tradition of phenomenology starting out with Edmund Husserl [17] and Martin Heidegger [18]. To my mind, this is the domain of selfhood, in addition to minimal and narrative, that needs to be focused upon to reach a firm understanding and definition of the suffering which affects persons living with chronic pain.

## The phenomenology of pain-related suffering

Pain is a feeling, an uncomfortable feeling, indeed. Feelings can be divided into three related types of phenomena: *sensations*, which make themselves known at certain locations in the body; *emotions*, which are about something in the world; and *attunements* or *moods*, which are not about particular things in the world, but rather determine the way the whole world appears to the person. The focus in more recent philosophy of feelings has primarily been on emotions rather than on sensations and moods, since the main point has often been to show how emotions are not merely bodily feelings but cognitive judgements about states of the world [19]. This is also reflected in the IASP definition of pain as “an unpleasant sensory and *emotional* experience” [3, emphasis added]. Phenomenologists, however, realized from very early on the importance of the other two aspects of human feelings: embodiment and world disclosure [20, 21]. It is not clear exactly what IASP intends by using the word “emotional” in their definition of pain, but, as I will try to show, it is primarily the attuned (‘mooded’) aspect of one’s experience that is brought into play in pain-related suffering.

Moods do not contain specific thoughts or perceptions in the same way that emotions do, but they nevertheless prefigure one’s general access to the world, as Martin Heidegger famously argued in his path-breaking work *Being and Time*, first published in 1927 [18]. Moods, also, as Matthew Ratcliffe has more recently pointed out, are, far from being devoid of bodily sensation, embodied in their very essence [21]. To feel anxiety or despair, for instance, are profoundly bodily experiences, as any sufferer from a panic attack or a depressive episode will know. Ratcliffe prefers the term “existential feelings” to the label of moods, but his central argument is that moods are bodily feelings *and* ways of finding oneself in the world simultaneously, and not accidentally so. The “lived body” is attuned and by this power also *world disclosing*, as Maurice Merleau-Ponty, another classic phenomenologist, pointed out in *The Phenomenology of Perception* [22]. The lived body (in contrast to the biological organism) is both the anchoring point of one’s existence in the world and the opening up of a meaningful space for one to exist in: the world as a space of possibilities.

To repeat, feelings, especially in the form of moods, are basic to one’s ‘being-in-the-world’ in Heidegger’s terminology, since they do the job of *opening up* the everyday world for us. To stress the focus on the what’s and why’s of the everyday world that one inherits from others and which change over time, notably by way of new types of activities, Heidegger’s teacher, Edmund Husserl, used the term “lifeworld” [17, part 3A]. One finds oneself *in* the (life)world, always already busy with different things that matter to the person, and this “mattering to” rests on an *attunement*, a mood-quality which the being-in-the-world always already has [18, paragraph 29]. Every activity is attuned in a way that brings out its significance, according to Heidegger. The different moods in question need not be powerful or directly paid attention to, but they are *there* as the constitutive ground of one’s being placed in the *meaning patterns of the world* (the lifeworld). Indeed, one does not choose the moods one finds oneself to be living in; the moods in question overwhelm and cannot easily be changed [18, paragraph 40]. Chronic pain is a good example of this.

Pain is a mood that is linked to one’s embodiment. A being without a body—a computer program, perhaps—is not, indeed, able to feel anything. Lacking a body, the computer/AI-program would not be attuned, and consequently it would not have any lifeworld, not “be-in-the-world” in the phenomenological sense [22]. Moods render the world meaningful, and this meaningfulness *feels* in the body (and in the world) [21]. That pain is not only a bodily sensation, but also a mood, means that pain permeates the entire embodied being-in-the-world of the afflicted person.

Pain as a mood, however, is not only world-*disclosing*, but also world-*enclosing* [23, 24]. In its most intense forms, pain forces one to focus *exclusively* upon one’s own body, which normally dwells in the background of one’s experience [25]. When it does so, as shown in the examples of overwhelming pain quoted above, the world literally disappears [26]. One’s horizon of meaningfulness gradually shrinks until nothing else, but the self-centred pain remains. Pain forces one by way of a centripetal movement to let everything else go and attain to it, only [27]. The lifeworld of the person in pain, consequently, is a world that is poorer than the one that preceded the onset of pain. The pain-mood makes it hard to engage in and focus on everyday projects; the meaning-patterns of the lifeworld do not open up in the smooth and accessible way as they did before. It takes a lot of energy to do anything else except focusing upon one’s own body, trying to endure and hold out.

Nevertheless, pain and the related suffering do not only concern the minimal domains of selfhood, but also what I have chosen to name the enworlded self. To show this more in detail, I will now turn to a published memoir of an author who endured and learned to cope with severe chronic pain. To focus upon such accounts is an alternative to doing interview studies, the way most pain researchers with a qualitative focus do. There exist many formally published (both online and in print) “pain diaries”—sometimes also known as “pathographies” which occasionally display stylistic qualities—as in the case below—which help one to understand the painful experiences the suffering person is undergoing [28]. To describe pain and the suffering it gives rise to is hard, since it tends to rob a person of her language and challenges the possibilities of everyday descriptions [29]. Even so, this one is quite convincing:

I am in pain from the moment I wake up until the moment I go to sleep. On a scale from 1 to 5, my pain never falls below 3 and will raise at some point to a 5 nearly daily and will stay there for hours. These spikes are unrelenting, stare-at-the-ceiling, wait-it-out pain. It sinews unfurling, the pain whips through the middle of my head. It feels like an ice-cream headache – the sharp, deep freeze of eating or drinking too much of a cold substance too quickly. It is heavier than ice-cream headache, though; it feels like an enormous weight lies along the tendon, crushing this central route, tearing my head in half. When spiking, the pain radiates out from the center and disperses. The ice cream has melted, not just freezing my forehead but dripping down to encompass my entire head symmetrically, pooling finally behind my eyes. I would later learn that in scientific terms, one could describe my pain even more accurately as “retro-orbital,” “holocranic,” “halocephalic”. In more emotional terms: Inescapable. Disabling. Punishing. Gruelling [30, chapter 8].

## The body broken

In *The Body Broken: A Memoire* Lynne Greenberg tells the story of her life and what might very amply be called her pain-related suffering (not using this concept, of course) [30]. At the age of nineteen, she fractures her neck in a grim car accident. After having undergone emergency surgery, installing a halo brace around her head, literally screwed into her cranium, joined to a corset to prevent her head from turning, and then spending a couple of months bedridden, the fissures on the upper part of her spine miraculously heal (very rare in the case of C2 vertebrae injuries). Subsequently, Greenberg is able to move on with her life and goes to university where she studies dance and art but in the end opts for a Master of Law. She marries a clever and kind man, Eric, who she meets in college, and has two children with him: Benjamin and Lilly, who are three years apart. Lynne and Eric both work as attorneys and make good money. The family lives in Brooklyn, enjoy a nice house in an idyllic neighbourhood, and have many friends and an extended family to support them. Life is good. Lynne spends a lot of time with her upgrowing children and other parents in the neighbourhood. On long maternity leave she also manages to finish a Ph.D. in English Literature and lands a job as a teacher at Hunter College in New York City, a mere seven subway stops from the place where she lives. She gives up her job as a lawyer—that she never liked much anyway—and is able to combine her academic career and major interest in life—seventeenth century British literature—with family life in Brooklyn.

Up to this point Greenberg’s story is mostly a fairy tale and all paradisaic. She also makes a point in her book of stressing that she is very privileged and lucky with what life has provided her with. But when Benjamin is fourteen and Lilly eleven, things start to happen. Life strikes back in the very form of her own previous back-bone injury:

Twenty-two years after my car accident, in the last days of summer 2006, I sat nervously eyeing a physician’s assistant. I had been walking around for weeks with head and neck pain, unable to move my head in any direction. Any movement of physical activity at all sent shock waves through the center of my head. I had treated these symptoms somewhat cavalierly at first, always assuming that my body would bounce back and that I would be fine, but my body had refused to do so. Starring at an MRI, the assistant finally announced, “Your neck is still broken.” And in that breath of moment, I could see that my life had fractured in two as clearly as had my fractured neck [30, chapter 2].

The metaphoric fracture she is talking about concerns the difference between her first 41 years of contentment and the succeeding years full of pain that will now start. She moves from paradise to hell, so to say, and, indeed, the classic work by John Milton, *Paradise Lost*, is often quoted in the book, alongside other great works of English literature (Greenberg’s speciality).

The sociologist Arthur W. Frank has focused on the storytelling of patients experiencing, exploring, and attempting to come to terms with life threatening diseases—like cancer or heart failure—and/or chronic diseases—such as kidney disease or diabetes. He identifies three types of stories that are typically consecutively formulated and told to others when being informed about such a medical condition: the *chaos* narrative, the *quest* narrative and the *restitution* narrative [31]. Firstly, one finds oneself in a situation of chaos, or even a state of denial; life does not make sense anymore and one simply does not understand what is happening. Secondly, one then organizes the whole life around questions connected to one’s medical condition, such as: ‘Why has this happened to me?’ ‘What is the true cause of my ailments in terms of an exact diagnosis?’ and ‘What are the treatment options and how can I get access to them?’. Thirdly, one finds a way of living with one’s medical condition. One builds a new life with a new identity that is worthwhile despite the remaining illness symptoms and a changed prognosis in terms of future risks and less expected life-years to come. When one has succeeded in doing so, one has moved on through the chaos- and quest narrative stages to a restitution narrative, a step one needs to take to be able to live a good enough life again.

The stories that Greenberg tells in her book fit this pattern very well, but whereas Frank’s terminology mainly seems to address what Cassell identified as attacks on the narrative self, what is falling apart in Greenberg’s case is more of her whole “worldly situation”, to put matters in phenomenological terms [5, 32]. The summer of 2006 Lynne has spent in London on a research stay, studying manuscripts in a library archive, trying to finish up a new book on seventeenth century literature she has been working on for three years. She has brought her children—but not her husband—and hired an au pair to look after Benjamin and Lilly, escorting them around London to museums, fairs, parks, cinemas, etc., during the days when she is in the library:

Two weeks into my work, I suddenly got a sharp, intense headache while working in the library. At first, I was so deep into the reading that I didn’t notice it, but as hours went by, it persisted. I had gotten to the library late that day and, focused on getting the work done, tried ignoring the pain, which didn’t seem like it could be such a big deal. I had never experienced a headache intense enough to hinder my work before. My eyes started burning and tearing from the effort to read through the headache. I kept squinting to see better but found the letters blurring and nearly impossible to read. I finally had to stop working; frustrated I vowed to get to work earlier the next day. In the morning, when I woke up, however, the headache was still there. It didn’t go away, and it has never gone away since [30, chapter 2].

This description of the onset of the intensive headache is very much in line with and like the classic piece on phenomenology of pain found in Jean-Paul Sartre’s *Being and Nothingness* [33, p. 444–445]. Just like in Sartre’s analysis, the pain enters the world of the pain sufferer gradually in and through the very activity of reading a book. But in the case of Greenberg, the pain has not gone away when she wakes up next morning—as I presume it did in the case of Sartre—but stays with her forever onwards. After having returned to New York with her children in August, Lynne becomes gradually unable to turn her neck and pick up things heavier than a thin book or newspaper—certainly not anything like the heavy manuscripts she had been studying in the library in London. This is a remarkable difference compared to the case found in Sartre. One of the first things that the unrelieving mood of pain does to Lynne’s life is to sabotage all her attempts to perform her academic work, not only the reading and writing itself, but also the teaching; she has to abstain from the upcoming autumn semester, ultimately giving her no choice but to resign from her position as a university professor.

Chronic pain that is intense and cumbersome enough most often does exactly this to the persons it afflicts. The pain-mood strikes at one of the most important meaning regions of the lifeworld (being-in-the-world) of the person: the structures of what I would like to call the “work-world” [34]. Working life may be more or less central to what a person identifies with and enjoys doing in her life, but it standardly occupies a more or less taken for granted component of everyday life that is turned upside down by intensive and long-lasting pain. The *enworlded self*, not only the narrative identity of the person, is therethrough dealt a blow by being refrained from performing the daily activities constituting working life habits and joys—in the case of Greenberg, reading, thinking, writing and teaching about literature. It may still be possible for Lynne to perform these activities to some extent and more occasionally—as reading and thinking is somewhat easier for her than writing and teaching—but not enough to keep up her profession.

The pain-related suffering of the enworlded self does not only concern working-life activities; pain also breaks havoc to family life meaning-structures. Because of the intense pain and difficulties to move, Lynne is no longer able to pick up the children from school, drive them to their after-school activities, do the grocery shopping, cooking, cleaning, and all other duties that belong to middle class American family life (for mothers mainly). Although her husband steps in and they also pay their long-term babysitter, Cora, to do more of the domestic services, Lynne feels deeply unsatisfied with the changed situation:

Performing domestic errands devolved as the condition failed to improve. For a while I managed to accomplish errands like picking up the dry cleaning in a haphazard way, but grocery shopping was simply impossible for me. (…) My former attempts at cooking (never much of a priority anyway) wholly dissipated. We did a brisk business with the neighbourhood restaurants that delivered, and Lilly and Benjamin ate endless meals of pizza and Chinese food. They ate alone on too many nights, since, as the fall progressed, I roused myself out of bed to eat dinner with them less and less frequently. On the rare conditions when I did join the kids for dinner, my attempts at discussion were routinely greeted with hostility, or, worse, intense sibling rivalry, as they competed for this tiny sliver of maternal attention [30, chapter 5].

Lynne feels she is losing control and doing damage to the whole family situation not only because of having to abstain from domestic duties (Eric does more than before, also, but commuting and having long working days in the city makes it complicated), but for failing to be around her children when they need her as a parent and best friend:

The relinquishing of control had several collateral effects – both financial and emotional. (…) The emotional ramifications of relying so much on Cora shifted almost schizophrenically from one day to the next. I vacillated between gratitude, relief, guilt, and frustration, knowing all the time that I had no real choice. Worse was the effect on my incapacitation on my relationship with Lilly. (…) I no longer had a close handle on the daily nuances, her subtle shifts of emotion, or negotiation with the outside world [30, chapter 5].

Alongside the previously mentioned fearfulness in relation to her medical condition and the pain itself, guilt and frustration are now moods that constantly permeates Lynne’s everyday world and existence. On top of worrying for Lilly and Benjamin, missing the close contact with them, and trying to shield them from the worrying prognosis of her condition, Lynne finally realizes that her new enworlded self simply excludes the possibility of being around children:

When Lilly came home from school, she had always run up the stairs to burst into my room. That fall, whenever she did so, she invariably woke me up from a nap. I now implemented a new rule. No more open-door policy. If I was in my room with the door closed, then the kids were not permitted to enter. They were no longer welcome. Mama had slipped, and, like Alice in Wonderland, was slipping farther and farther away from them, down the rabbit hole. I just couldn’t figure out how to stop tumbling [30, chapter 5].

Another thing that Greenberg notices is that the pain makes it harder for her to concentrate on and remember practical things. She forgets to pick up Lilly, to pay the bills, to respond to e-mails, to turn up to arranged meetings with friends, or, more often, the friends catch her unguarded at home since she has forgotten about inviting them. She also has difficulties finding important items in her home, like keys, pieces of clothing, credit cards or books, since the pain makes her no longer capable of paying attention to her surroundings and remember where she has put them. Lynne’s memory is also affected in the way that she has difficulties remembering about specific events in the past, everything is somehow “foggy” to her (mood-description again). Increasingly she also finds it difficult to recognize neighbours living close by and their names:

I saw her rushing down the sidewalk one evening looking quite distressed. When I asked her if there was anything wrong, and if I could help, she replied, obviously quite upset, “Mike just got into a car accident and is at the hospital”. I looked at her blankly. “Who is Mike?” I asked. “My husband,” she nearly shrieked and kept running. Of course, I knew his name. It had, at that moment, however, completely evaporated. Finding me so unforgivably lame, she called upon other neighbours and friends that night to help her, and for that, I feel both remorseful and helpless. So much for helping someone else for a change [30, chapter 31].

Of course, the damage is done to Lynne’s narrative self (her self-image), as well, but it is the enworlded self that is experiencing this very failure to think and act according to established meaning patterns. It is the enworlded self that no longer feels its way forward in a firm way, navigating through the terrain of acquaintances and relationships that used to fill up Lynne’s worldly existence when it was properly attuned. Suffering from chronic pain can in this sense resemble cases of mild cognitive impairment (MCI), or even dementia.

## The patient-world

Greenberg’s headache and inability to move her neck and carry things around escalate during the autumn and wintertime of her first half of a year with the medical condition. The worldly domains of working life and family life (including friends, travels, hobbies, etc.) are shrinking down to an absolute minimum:

By December, I barely got out of bed at all. I had come to a point where I found it easier to just lie immobile than try to walk around or move, as physical activity always increased the pain. I rarely left the house, except to go to the doctor for shots. I had evolved, without clearly realizing it, to an invalid, lying prone with a long thin pillow slid under my neck, my bedside table laden with bottles upon bottles of pills. (…) Pain penetrated every mite of concentration, infiltrating in a full-frontal attack, a D-day to the body. It left me lethargic, dolorous; all I wanted to do was lie there. I was so preoccupied with my beached body that I allowed my mind to become a desert as well (…) all thoughts monomaniacally revolved around my physical condition [30, chapter 6].

This situation and experience resemble the cases of overwhelming pain, afflicting the minimal self, that I quoted from above [8]. But the pain that Greenberg records is not always as intensive as that, and, when it is so, only for brief periods. These “full-frontal attacks”, however, leave her exhausted and unable to *enworld* into the physical and mental territories beyond her bedroom door. This means that the overwhelming pain has an enduring effect on her enworlded (and also narrative) self as soon as the minimal self allows the rest of her existence to, so to say, appear on stage again. The lived body is anchored in the minimal self but it stretches out into the enworlded aspects of selfhood that becomes visible by way of Greenberg’s descriptions.

One more thing is quite striking in the quote from Greenberg above: the references to doctors, shots and pills, and the obsessed focus upon the medical condition of her own body. Persons who fall ill generally get preoccupied with the working mechanisms of their bodies on an anatomical, physiological, and biomedical level. But if the medical diagnosis and prognosis of what they are suffering from is uncertain or unknown—as is often the case for patients with chronic pain conditions—this preoccupation easily develops into a full-time job or even an obsession. To search for information about possible diagnoses and treatment options, to try to get appointments with the best physicians and physiotherapists, to get into the best hospitals and clinics, to deal with health insurance managers, all this takes up aeons of time. There is not much time left for other things to do and think about when the pain has transitioned from unbearable to manageable again for the time being.

If the enworlded self is broken, restricted, and alienated by chronic pain, it is so mainly in the domains of the meaning-structures of the lifeworld that refer to work and family life, including, of course, close-others outside the family and further interests than the ones represented by one’s profession [16]. Following Alfred Schutz, one could say that the meaning-structures of the lifeworld can be divided into several different partial and overlapping regions: the “work-world” and the “family-world” are the ones I have concentrated upon so far [34]. If the work-world and the family-world are broken or even closed down by the experience of chronic pain, one should also add that a new territory of the lifeworld appears to open up, the “patient-world” [35, 36].

Susan Sontag once wrote, in a famous passage, that all persons belong to two different worlds from the very start of life, but that:

Illness is the night side of life, a more onerous citizenship. Everyone who is born holds dual citizenship, in the kingdom of the well and in the kingdom of the sick. Although we all prefer to use the good passport, sooner or later each of us is obliged, at least for a spell, to identify ourselves as citizens of that other place [37, p. 3].

What I call the patient-world is very much like that which Sontag names the kingdom of the sick. Except the sick are not kings in this world, other persons are, persons who have not yet checked out from the kingdom of the well: health-care professionals and insurance administrators, mainly. The patient world is consequently full of strategic considerations and tactic moves in dealing with such professionals to find the way to the most beneficial (and presumably correct) diagnosis and treatment in order to be able to use the good passport again (at least temporarily).

Most of the chapters in Greenberg’s book are about navigation in the patient-world. About different drugs that may ease the pain, but always at the cost of sideeffects and possible addiction. About physiotherapy, acupuncture, injections, electric massage, surgery, rehab-programs and many other medically-related things that could possibly if not cure so at least alleviate her condition. She learns a lot about neck pain as a result of damage done to the anatomical structure of the C2 vertebrea, but also about the whole-body anatomy and physiology of pain, including biomolecular processes, as a result of her encounters with representatives of various specialities and professions: pain anaesthesiologists, neurosurgeons, sports medicine doctors, endocrinologists, internists, physiotherapists, chiropractors, osteopaths, kinesiologists, and others. Still, she remains an amateur in the game and has to rely upon advice from friends who are more knowledgeable and the information she can find in journals and on the internet (the possibilities today have grown immensely compared to what was the case in 2006). Her husband, Eric, takes on a large workload in her patient-world, despite still remaining among the healthy, and devotes much of his free time to make telephone calls to health insurance administrators, make appointments with pain specialists and compare published results regarding various treatment options and rehab programs for her.

Finally, Lynne agrees to have surgery attempting to fuse the C1 and C2 vertebrae to stabilize her neck and ease the pain. Unfortunately, this turns out to be a rather bad idea. The pain does not get better and other problems emerge as a result of the operation: difficulties to focus eyesight and opioid dependency, mainly. During springtime 2007 her family-world is further challenged by economic problems having to do with all the consultations and treatments (as health care in the USA is not cheap even with excellent insurance) and not being able to control escalating household costs. Lynne gets depressed and starts having fantasies about killing herself, since she feels like life is unbearable and meaningless anyway. Finally, she is accepted into the program of a headache-focused clinic which offers new hope. By way of the daily schedule and gradually getting to know her fellow patients, new avenues of the patient-world open up for her. A fellowship of pain-related sufferers is formed in the kingdom of the sick of the hospital clinic. The patients share stories, comfort each other and form solidarity bonds; they know what it is like to have “inescapable, disabling, punishing and gruelling pain” (see above), as nobody else does:

People’s stories, for all our individual idiosyncrasies, shared a common theme: we felt that our struggles with pain had severely compromised our work and family lives in ways that healthy people cannot and do not understand. It was a relief to be among the like-bodied and like-minded. We had all been away, pillaged by pain. There was no need to explain myself, my level of misery, suffering, exhaustion, or body loathing; everyone there was living the same crippled and crippling existence [30, chapter 25].

In this paragraph, Greenberg confirms the phenomenological analysis of the two regions of the lifeworld which are being ‘severely compromised’ by chronic pain: ‘work and family lives,’ representing the damage done to the work-world and the family-world suffered by the enworlded self in my terminology. She also, however, indicates what a richer patient-world could look like, finding a way out of the loneliness and despair by forming bonds with other patients. Lynne realizes that other persons in the clinic have even worse symptoms and problems in their lives than she does. Even if she could not help her neighbour with the husband, whose name she had forgotten, she may be able to help them by being attentive and empathic. This will also make her less self-focused:

Meeting other people with similar problems had a healing effect upon me. I no longer felt so alone or unique. I realized that others shared my most intimate problems and had found their lives equally impacted by pain. The hospital catapulted me out of my seclusion, and I thereafter felt differently toward my situation. I began to learn a bit of compassion, and it permanently shattered my previously ingrained patterns of narcissism [30, chapter 25].

Being told by the chief neurologist in the clinic that her previous operation was actually necessary and lifesaving because of the instability of her neck, Lynne recovers hope and is able to get out of the depressed mood that has been haunting her. A new life is opening up, not without pain, but manageable and shared with others, including not only other chronic pain patients, but also family and friends. She has moved on from the chaos and quest narratives to a restitution narrative, to put it in the terminology of Frank [31]. This movement, however, has been performed not chiefly by the narrative self but by the enworlded self, changing concrete worldly meaning-patterns of living, not only abstract self-understanding. The changed patterns of life—finding new meaning-structures to move along and follow in the lifeworld (her being-in-the-world)—concern everyday actions but also changed relationships to the persons who are most important in Lynne’s life. This includes losing or giving up some old relationships and establishing new ones with persons that share or understand what it is like to live with chronic pain.

Implementing such changes in everyday action and intersubjectivity patterns, in combination with finding ways to control the feelings of the minimal self by way of cognitive and physical strategies, Greenberg is finally able to change her mood(s). Her everyday attunement and being-in-the-world attain a more homelike quality again in contrast to the fundamental alienation that has dominated her existence the last year [32]. Pain is still a part of her world experience and some days it overwhelms her, but it no longer controls and alienates her existence to a degree which makes it impossible to live a good (enough) life [38].

## Conclusion

In the research and literature on pain-related suffering in disciplines such as philosophy of medicine, psychology, palliative medicine, medical ethics, nursing, sociology, psychiatry and gerontology, a difference has recently been pointed out between suffering processes having to do with experiences of the minimal self in contrasts to threats to the integrity and intactness of what is known as the narrative self (also being referred to as a person). I have proposed that in addition to these two, an in-between layer of selfhood needs to be distinguished and studied to understand pain-related suffering: the enworlded self and its mood-related ways of inhabiting versus failing to inhabit the lifeworld.

Pain-related suffering is a complex phenomenon involving mood-related processes and everyday challenges on at least three different levels: bodily experiences, lifeworld matters and questions about personal identity. These three different levels correspond to three connected aspects of layered selfhood: the minimal self, the enworlded self, and the narrative self. In order to have an enworlded self, a person must have a minimal self, but in order to also have a narrative self, the enworlded aspects are absolutely crucial and necessary. By way of using a published memoire focused on chronic pain experiences, I have tried to show that this is the case, and also identified two different regions of the lifeworld in which the damaging and alienating experiences are taking their toll: the work-world and the family-world.

In addition to this, a third region of the lifeworld has been identified and named as the patient-world, a region which is formed and expands as the chronic-pain sufferer searches for medical-professional advice and assistance. The patient-word is also an unhomelike and alienating terrain, but it could be made more homelike by forming bonds with fellow chronic-pain sufferers and finding professionals who one trusts. From the point of view of health-care professionals and other people trying to assist and help persons experiencing pain-related suffering, it is crucial to focus on all three layers (levels) of selfhood distinguished above and understand how they relate to different forms of alienating moods. In addition to the pain mood itself, moods that involve losing control, feeling fearful, sad or desperate in relation to the pain and the regions of the lifeworld that it destroys or limits access to, are involved. By addressing the three different layers of selfhood and how they interact in the everyday life of the patient, chronic-pain suffering can be better understood and, also, possibly alleviated by taking appropriate measures that fit the attuned pattern of the pain experiences of each individual case.
